# Genotypic Characterization of *Yersinia enterocolitica* Biotype 4/O:3 Isolates from Pigs and Slaughterhouses Using SE-AFLP, ERIC-PCR, and PFGE

**DOI:** 10.1155/2013/521510

**Published:** 2013-05-30

**Authors:** Renata Paixão, Luisa Zanolli Moreno, Débora Dirani Sena de Gobbi, Daniele Cristine Raimundo, Thais Sebastiana Porfida Ferreira, Maria Garcia Spindola, Ernesto Hofer, Cristhiane Moura Falavina dos Reis, Maria Helena Matté, Andrea Micke Moreno

**Affiliations:** ^1^Departamento de Medicina Veterinária Preventiva e Saúde Animal, Faculdade de Medicina Veterinária e Zootecnia, Universidade de São Paulo, Avenida Prof. Dr. Orlando Marques de Paiva 87, Butantã, 05508-270 São Paulo, SP, Brazil; ^2^Faculdade de Medicina Veterinária, Faculdades Metropolitanas Unidas (FMU), Rua Ministro Nelson Hungria 541, 05690-050 São Paulo, SP, Brazil; ^3^Laboratório de Saúde Pública, Faculdade de Saúde Pública, Universidade de São Paulo, Avenida Dr. Arnaldo 715, 01246-904 São Paulo, SP, Brazil; ^4^Laboratório de Zoonoses Bacterianas, Fundação Instituto Oswaldo Cruz (FIOCRUZ), Avenida Brasil 4365, 21045-900 Rio de Janeiro, RJ, Brazil

## Abstract

*Yersinia enterocolitica* is a foodborne pathogen that causes illness in humans and animals. The biotype 4/O:3 has been commonly associated with yersiniosis and is characterized by the presence of chromosomal and extra-chromosomal virulence genes. Molecular typing methods have been successfully used to characterize *Y. enterocolitica* genetic heterogeneity and to study the epidemiology of the bacteria from different origins. In this study, 320 *Y. enterocolitica* biotype 4/O:3 isolates originating in pigs and slaughterhouses were characterized according to the virulence profile, and 61 isolates were typified through SE-AFLP, ERIC-PCR, and PFGE techniques. The majority of the isolates originated from pigs, and the predominant virulence profile was *ail*+ *virF*+ *rfbC*+ *ystA*+, representing 83.4% of the tested isolates. All of the *Y. enterocolitica* 4/O:3 isolates were positive for at least *ystA* gene. The SE-AFLP and ERIC-PCR patterns were highly homogeneous. The SE-AFLP was more discriminative than the ERIC-PCR and tended to cluster isolates according to the slaughterhouse. Despite the limited genetic diversity of *Y. enterocolitica* 4/O:3, PFGE was shown to be the most discriminative technique considering one band of difference. Fattening pigs proved to be an important reservoir of *Y. enterocolitica* biotype 4/O:3 carrying virulence genes.

## 1. Introduction

*Yersinia enterocolitica* is an important zoonotic foodborne pathogen that can cause acute diarrhea, terminal ileitis, and mesenteric lymphadenitis in humans and animals [1, 2]. The isolates largely responsible for human *yersiniosis* in Europe, Japan, Canada, and the USA belong to biotype 4/O:3 [1]. The epidemiology of the disease is not completely known. 

The pig is considered the only reservoir from which pathogenic *Y. enterocolitica* isolates, such as biotype 4/O:3, have been frequently isolated [1]. The prevalence of this biotype in pig slaughterhouses has been reported to be 56% in Finland [3] and 60% in southern Germany [4]. Serotype O:3 is predominant among the isolates recovered from slaughter pigs in the USA [5]. Martínez et al. [6] reported that fattening pigs seem to be an important reservoir of pathogenic *Y. enterocolitica* in Belgium, Italy, and Spain.

The virulence of the pathogenic biotype 4/O:3 is attributed to the presence of chromosomal and extrachromosomal genes. The plasmid for *Yersinia *virulence (pYV) encodes the adhesin A (*YadA*), the *Yersinia *outer proteins (*Yops*) from type III secretion system, and the transcriptional regulator gene (*virF*) [7, 8]. The chromosomal virulence genes include *inv* (invasin), *ail* (attachment and invasion locus), and *ystA* (*Yersinia* stable toxin A) [9]. A number of these factors are restricted to the pathogenic pYV-bearing strains of *Y. enterocolitica* such as *ail* and *ystA,* while the *inv* gene is common to pathogenic and nonpathogenic strains [10]. 

Molecular typing methods have been used to study the genetic heterogeneity of *Y. enterocolitica*. Much of the data obtained, especially those regarding serotype O:3, indicate that these strains are well conserved [11]. Despite the limited genetic diversity, *Y. enterocolitica* biotype 4/O:3 strains have been successfully characterized by typing methods such as Repetitive Extragenic Palindromic (REP) and Enterobacterial Repetitive Intergenic Consensus Sequences-(ERIC-) PCR, PCR-ribotyping, and Pulsed Field Gel Electrophoresis (PFGE) [10–14].

In Brazil, the epidemiology of *Y. enterocolitica* is not commonly studied. In one of the few studies about the bacterium, Falcão et al. [10] reported the high similarity of human and animal isolates pulsotypes. These PFGE results corroborate previous observations from other countries, and it was the first time that pigs were established as a potential source of *Y. enterocolitica* 4/O:3 human infection in Brazil. The aim of this study was to characterize *Yersinia enterocolitica* 4/O:3 isolates originating in pigs and the environment of slaughterhouses in Sao Paulo, Brazil, by Amplified Fragment Length Polymorphism with a single enzyme (SE-AFLP), ERIC-PCR, and PFGE techniques.

## 2. Materials and Methods

### 2.1. Culture Collection Strains

The following strains were used as positive and negative controls for biochemical and PCR tests: *Yersinia enterocolitica *O:3 biotype 4 (MyO—SW/897/63), *Y. enterocolitica *O:8 biotype 1B (P311—WF—Albany, NY USA), *Y. enterocolitica *O:9 biotype 2 (My79—Nilhén—Suécia), *Y. pseudotuberculosis*—IAL1791, *Y. frederiksenii*—CIP8029 and *Y. kristensenii*—CIP9993. These strains were supplied by Dr. Ernesto Hofer from the Bacteriology Department of the Oswaldo Cruz Institute, RJ, Brazil (IOC/FIOCRUZ).

### 2.2. Sampling and Microbiological Analysis

A total of 320 *Y. enterocolitica* biotype 4/O:3 isolates were studied. These isolates were previously obtained, as described by Paixão et al. [15], in tonsils, swabs from slaughterhouses and in market environment points, and pork gathered from 12 collections conducted between 2007 and 2008. 

The samples were processed with cold enrichment with phosphate-buffered saline, sorbitol 1%, and bile salts 1.5% (Difco-BBL, Detroit, MI, USA) for 10 to 14 days. The 14-day enrichment was followed immediately by an alkali treatment with 0.5% KOH in 0.5% saline solution for 20 seconds before plating on a selective agar plate. An aliquot of the broth (10 *μ*L) was plated onto MacConkey (Difco-BBL, Detroit, MI, USA) and Cefsulodin-Irgasan-Novobiocin (CIN) agar (Difco-BBL, Detroit, MI, USA). The plates were incubated for 24 h at 30°C, under aerobic conditions. The colonies presenting suggestive morphology were selected from each selective agar for biochemical identification with Kligler iron and Christensen urea tests and fermentation of sucrose, rhamnose, and melibiose.

The isolates positive for biochemical identification were biotyped according to the biotyping schema proposed by Souza et al. [16], and they were serotyped using monovalent antiserum agglutination tests in the Bacteriology Department of the Oswaldo Cruz Institute, RJ, Brazil (IOC/FIOCRUZ).

### 2.3. DNA Extraction and Virulence Genes Detection

Purified DNA was recovered according to Boom's et al. [17] protocol of DNA extraction and stored at 20°C. The DNA samples were amplified by simultaneous detection of the *ail* gene for the attachment invasion locus; the invasin gene (*inv*), the *Yersinia* heat stable enterotoxin A gene (*ystA*), the O:3-antigen gene (*rfbC*), and the virulence-regulatory factor gene (*virF*), as described by Thisted Lambertz and Danielsson-Tham [18]. The amplification was carried out in a 50-*μ*L reaction mixture that contained 5 *μ*L of DNA template, 1.5 mM of MgCl_2_, 200 mM of each dNTP, 20 pmol of each primer and 1 U of Taq DNA polymerase (LGC Biotecnologia, São Paulo, Brazil), 1 X PCR buffer, and ultra-pure water. 

The amplification conditions were as follows: an initial denaturation at 94°C for 3 min, followed by 30 cycles of denaturation at 94°C for 30 sec, annealing at 60°C for 1 min, and extension at 72°C for 1 min, with a final extension at 72°C for 5 min. The PCR products were separated in 2% agarose gel stained with BlueGreen (LGC, São Paulo, Brazil), and they were identified using a 100 bp DNA ladder (LGC Biotecnologia, São Paulo, Brazil).

### 2.4. Genotyping

From the 320 *Y. enterocolitica* biotype 4/O:3 isolates, 61 were selected for genotyping using the SE-AFLP, ERIC-PCR, and PFGE techniques. The strain selection was based on the slaughterhouse of origin, the collect number, and the positive animal.

#### 2.4.1. Single Enzyme Amplified Fragments Length Polymorphism (SE-AFLP)

SE-AFLP protocol was performed according to the description of McLauchlin et al. [19], and its products were detected with electrophoresis at 28 V for 24 h in 2% agarose gel stained with BlueGreen (LGC Biotecnologia, São Paulo, Brazil) The images were captured under UV illumination using the ImageMaster Photo Documentation System (GE Healthcare do Brasil Ltda, São Paulo, Brazil).

#### 2.4.2. Enterobacterial Repetitive Intergenic Consensus Sequences (ERIC-PCR)

The ERIC-PCR was performed as described previously by Versalovic et al. [20], using 5 *μ*L from extracted DNA, 1.5 mM of MgCl_2_, 10 pmol from primers ERIC1 (ATGTAAGCTCCTGGGGATTCAC), and ERIC2 (AAGTAAGTGACTGGGGTGAGCG), 1.0 U of Taq DNA polymerase (LGC Biotecnologia, Sao Paulo, Brazil), 1 X PCR buffer, and water to complete the total volume of 50 *μ*L. The PCR reaction was conducted with 35 cycles, consisting of denaturation for 4 min at 94°C, annealing for 60 sec at 50°C, and extension for 2.5 min at 72°C, which increased with a final extension for 20 min at 72°C, as described by Rafiee et al. [21]. The amplification products were detected through electrophoresis at 35 V for 20 h in 2% Agarose 1000 (Invitrogen Corporation, Carlsbad, CA, USA) gel stained with BlueGreen (LGC Biotecnologia, São Paulo, Brazil).

#### 2.4.3. Pulsed Field Gel Electrophoresis (PFGE)

The colonies were transferred to 10 mL of BHI broth (Difco) and grown overnight at 28°C under shaking. The bacterial suspension was centrifuged at 1500 rpm for 10 min, the supernatant was discarded, and the pellet was resuspended in 10 mL of PETT IV solution (10 mM Tris-HCl [pH 8.0] and 1 M NaCl, 10 mM EDTA). The bacterial suspension was recentrifuged at 1500 rpm for 10 min, the supernatant was discarded, and the pellet was resuspended in 1 mL of lysis buffer (1 M NaCl, 10 mM Tris [pH 8.0], 200 mM EDTA, 0.5% sarcosyl, and 0.2% sodium deoxycholate). Agarose Seakem gold 2% (Cambrex Bio Science Rockland, Inc., East Rutherford, NJ, USA) was prepared in 0.5 X TBE. A volume of 400 *μ*L of the bacterial suspension was heated to 40°C, and it was added to 400 *μ*L of heated 2% agarose solution. The mixture was immediately dispensed into wells and chilled for 10 min at 8 to 10°C. Plugs were placed into 2.5 mL of Lysis buffer, and 70 *μ*L of proteinase K (0.5 mg/mL; LGC Biotecnologia, Sao Paulo, Brazil) was added before incubation at 56°C for 20 h. The plugs were rinsed once in 1 mL TE buffer (10 mM Tris, 1 mM EDTA).

The plugs were washed with 5 mL of 1 X TE buffer twice for 30 min each time at 37°C, and they were then stored in 1 mL of 1 X TE buffer at 4°C. The DNA was cleaved with 5 U of *Not*I enzyme (New England BioLabs) per *μ*g of DNA for 4 h at 37°C. PFGE (CHEF-DR III; Bio-Rad Laboratories, CA, USA) was conducted in a 1% Seakem gold agarose gel (Cambrex Bio Science Rockland, Inc., East Rutherford, NJ, USA) in 0.5 X TBE buffer, with a switching time of 7 to 23 sec for 45 h at 14°C. Gel was stained with 1 X SybrSafe (Invitrogen Corporation, CA, USA) for 40 min and photographed under UV transillumination. DNA fragments were identified using Lambda DNA-PFGE marker (New England BioLabs Inc., USA).

### 2.5. Statistical Analysis

The levels of relatedness of the isolates were determined through a comprehensive pairwise comparison of restriction fragment sizes, using the Dice coefficient. The mean values obtained from the Dice coefficients were applied in UPGMA, using BioNumeric 6.6 (Applied Maths) to generate dendrograms. 

A 90% cut-off was used to analyze the clusters generated by AFLP and ERIC-PCR techniques. Samples that had four or more different bands were classified into different pulsotypes [22]. The discriminatory index was calculated using at least one band of difference among the isolates, as described by Hunter and Gaston [23].

## 3. Results

Of the 320 *Y. enterocolitica* biotype 4/O:3 isolates, only two (0.63%) were isolated from the Slaughterhouse 1 environment. The remaining isolates originated from tonsil samples from pigs of both slaughterhouses. There were no isolates from pork and Slaughterhouse 2 or the market environments. The virulence profiles obtained through PCR testing of the 320 *Y. enterocolitica* 4/O:3 isolates are described at Table 1. The predominant profile was *virF*+ *ail*+ *rfbC*+ *ystA*+, representing 83.4% of the tested isolates and having a similar high frequency in both slaughterhouses. The *virF*-* ail*+ *rfbC*+ *ystA*+ profile was the second most observed incident virulence pattern in both slaughterhouses. All *Y. enterocolitica* 4/O:3 isolates were positive for at least the *ystA* gene; both environmental isolates were positive for all tested virulence genes. 

**Figure 1:**
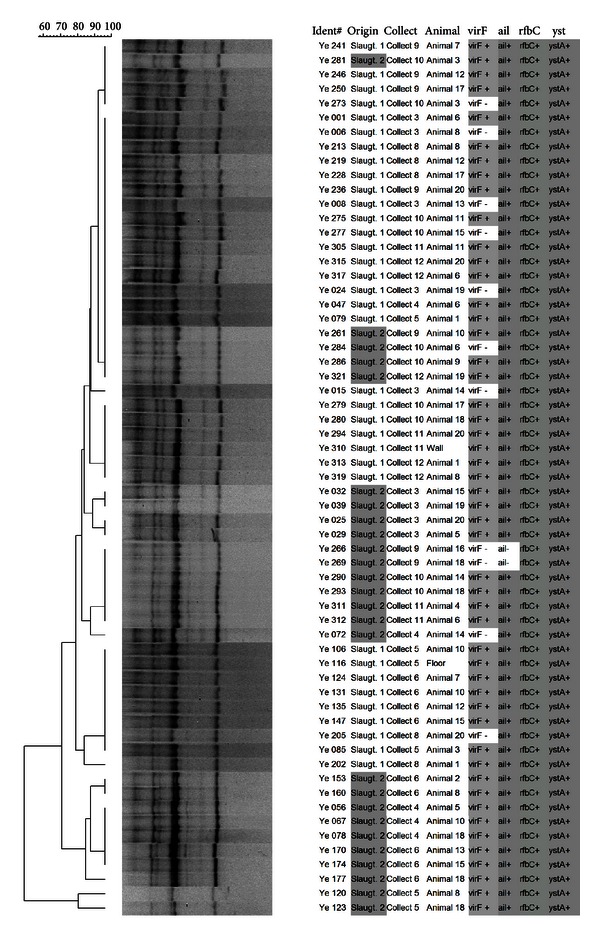
Dendrogram showing comparison of *Y. enterocolitica* biotype 4/O:3 isolates by SE-AFLP.

**Figure 2:**
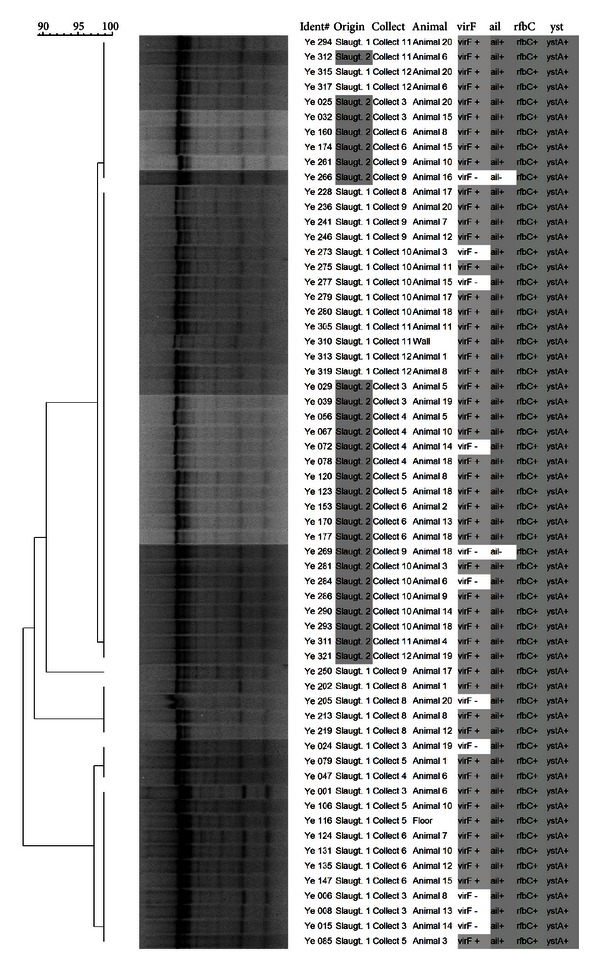
Dendrogram showing comparison of *Y. enterocolitica* biotype 4/O:3 isolates by ERIC-PCR.

From the 320 isolates, 61 were selected for genotyping by SE-AFLP, ERIC-PCR, and PFGE. The analysis of AFLP dendrogram (Figure 1) revealed that 15 patterns were highly homogeneous. Nine clusters were identified using a 90% cut-off; it was observed that the isolates tended to be grouped according to slaughterhouse, and a clustering tendency related to the virulence profile was not observed. The ERIC-PCR patterns were more conserved (Figure 2), and the 90% similarity cut-off resulted in only two clusters (A and B). Cluster A was composed of 77.05% of *Y. enterocolitica* isolates that presented four distinct ERIC-PCR patterns. A clustering tendency was not observed among the genotypes related to the origin, isolation site, or even virulence profile. The discriminatory indexes obtained for the AFLP and ERIC-PCR were, respectively, 0.70 and 0.36.

**Figure 3:**
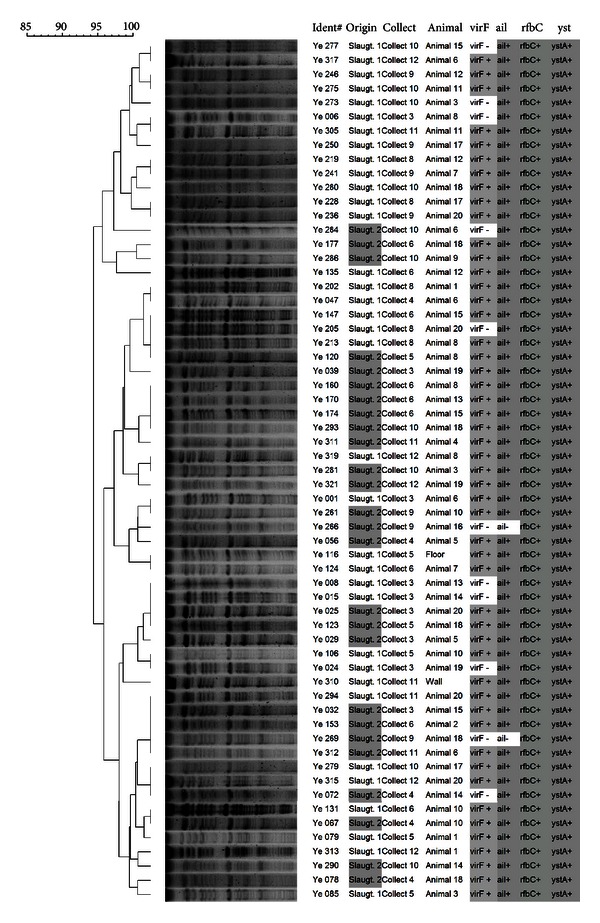
Dendrogram showing comparison of *Y. enterocolitica* biotype 4/O:3 isolates by PFGE.

The PFGE dendrogram (Figure 3) revealed 35 profiles. It was observed that the isolates tended to be grouped according to slaughterhouse; this finding was not sustained among all of the profiles. The virulence profiles presented a similar condition; they were not clustered in the same profiles, although they presented a stronger tendency to be grouped with isolates of the same origin and month of collect. The discriminatory index obtained for the PFGE techniques was 0.97; however, using the criteria suggested by van Belkum et al. [22], all of the tested isolates formed only one cluster or pulsotype. 

## 4. Discussion

In this study, the application of three molecular typing methods, AFLP, ERIC-PCR, and PFGE, was used to characterize *Y. enterocolitica* biotype 4/O:3 isolates. The virulence profile was also characterized by studying the *ail*, *ystA*, *rfbC*, and *virF* genes. Most of the isolates originated from pig tonsil samples and presented all of the virulence genes studied, suggesting that fattening pigs are an important reservoir of *Y. enterocolitica* biotype 4/O:3-carrying virulence genes in Sao Paulo State slaughterhouses.

*Yersinia enterocolitica* is an important enteric pathogen; the major routes of infection are presumed to be foodborne, and pork is one potential source of human infection [1, 4]. In Europe, *Y. enterocolitica *biotype 4/O:3 has been shown to be predominant among asymptomatic pigs [24]. The prevalence of biotype 4/O:3 in slaughtered pigs has been reported to be higher than 50% in Finland and Germany [3, 4]. 

Our results corroborate these previous data, showing a higher prevalence of *Y. enterocolitica *biotype 4/O:3 in pig and no isolation of this biotype in pork or the meat market environment. Our results also contradict the classical knowledge of pork as an important source of human infection [1, 25]. The high isolation rates of *Y. enterocolitica *biotype 4/O:3 in farm and slaughtered pigs are an important public health issue, particularly when they are reservoirs of *Y. enterocolitica*-carrying virulence genes.

More than 80% of the studied isolates presented the virulence genes *ail*, *ystA*, *rfbC*, and *virF*, and all of the isolates were positive for at least *ystA* gene. The only genes that presented variable results were *virF*, *rfbC*, and *ail*. The transcriptional regulator gene (*virF*) is an extrachromosomal virulence gene whose presence is attributed to pathogenic biotypes [7]. Falcão et al. [10] reported a variable incidence of *virF* gene in *Y. enterocolitica* isolates of animal origin and variable results of plasmid genes expression. 

The chromosomal *ail* gene has been described as a virulence marker that has a high correlation with virulent *Y. enterocolitica* [26]. Because of its stability as a virulence marker, *ail* has been used as detection method for pathogenic *Y. enterocolitica*. Tadesse et al. [27] reported the *Y. enterocolitica* serogroup O:3 isolates negative for the *ail* gene. In this study, only ten isolates presented the virulence profile *virF*- *ail*- *rfbC*+ *ystA*+. The *rfbC* gene is known as a marker for pathogenic *Y. enterocolitica* biotype 4/O:3 [18, 28]. Limited information is available in the literature about this gene, and as described for *virF* and* ail*, little is known about the impact of the absence of these genes or the lack of their expression in the virulence of *Y. enterocolitica* biotype 4/O:3. 

The molecular typing of *Y. enterocolitica* 4/O:3 through AFLP, ERIC-PCR, and PFGE showed high homogeneity, with more than 80% similarity among the 61 isolates tested in this study. The DNA-based molecular methods have been successfully used to characterize *Y. enterocolitica* biotypes and serotypes [10, 29]. The biotype 4/O:3 isolates present limited genetic diversity. Our results illustrate this fact, particularly in the ERIC-PCR analysis; although this technique has been considered more discriminating than REP-PCR, it appears to be more useful for separating the isolates among the different serotypes [11, 13]. 

The AFLP presented a higher discriminatory index than the ERIC-PCR, and it was observed that the isolates tended to be grouped according to the slaughterhouse of origin. This technique appears to better differentiate *Y. enterocolitica* according to the biotypes and serotypes, and the biotype 4/O:3 isolates are considered the most clonal [27, 29]. 

PFGE was the best technique to differentiate *Y. enterocolitica* biotype 4/O:3 isolates considering one band of difference, but all of the isolates presented similarity higher than 91% and less than four bands of difference. The homogeneity of the biotype 4/O:3 pulsotypes has been reported [12, 25], although it was shown that the use of three macrorestriction enzymes could efficiently differentiate biotype 4/O:3 isolates in distinct pulsotypes [14, 30]. The use of one enzyme to macrorestriction analysis proved to be useful and more practical for laboratory routine analysis [10].

Even though molecular typing methods appear to evaluate clonality among *Y. enterocolitica* biotype 4/O:3 isolates, little information is available regarding if a clustering tendency related to origin, isolation site or even virulence profile exists. Consequently, these methods have limited benefits in epidemiological studies [24]. Therefore, DNA-based typing techniques must be supplemented with additional epidemiological information and both pheno- and genotypic characterization to enhance the knowledge of *Y. enterocolitica* biotype 4/O:3 isolates. 

## Figures and Tables

**Table 1:** Virulence gene profiles obtained through PCR on *Yersinia  enterocolitica  *biotype 4/O:3 isolates.

Virulence gene profile				General	Slaughterhouse 1	Slaughterhouse 2
*virF*+	*ail*+	*rfbC*+	*ystA*+	267 (83.4%)	148 (80.8%)	119 (87.0%)
*virF- *	*ail*+	*rfbC*+	*ystA*+	40 (12.5%)	31 (17.0%)	9 (6.5%)
*virF- *	*ail- *	*rfbC*+	*ystA+ *	5 (1.6%)	0 (0.0%)	5 (3.5%)
*virF- *	*ail- *	*rfbC- *	*ystA+ *	8 (2.5%)	4 (2.2%)	4 (3.0%)

				320 (100%)	183 (100%)	137 (100%)
